# In-season eccentric-overload training in elite soccer players: Effects on body composition, strength and sprint performance

**DOI:** 10.1371/journal.pone.0205332

**Published:** 2018-10-16

**Authors:** Luis Suarez-Arrones, Eduardo Saez de Villarreal, Francisco Javier Núñez, Valter Di Salvo, Cristian Petri, Alessandro Buccolini, Rafael Angel Maldonado, Nacho Torreno, Alberto Mendez-Villanueva

**Affiliations:** 1 Department of Sport and Informatics, Section of Physical Education and Sport, Pablo de Olavide University, Sevilla, Spain; 2 Football Performance & Science Department, ASPIRE Academy, Doha, Qatar; 3 Department of Movement, Human and Health Sciences, University of Rome “Foro Italico”, Rome, Italy; 4 Sports and Exercise Medicine Unit, Department of Experimental and Clinic Medicine, University of Florence, Florence, Italy; 5 Sport Sciences Department, ACF Fiorentina S.p.A., Florence, Italy; Universidad Europea de Madrid, SPAIN

## Abstract

The aim of this study was to describe the changes in body composition, strength and sprint performance in response to an entire competitive season of football training supplemented with 2 inertial eccentric-overload training sessions a week in young male professional soccer players. Whole body and regional composition (assessed using dual-energy X-ray absorptiometry), power output in half-squat and 40-m sprinting performance were evaluated in fourteen players. The eccentric-overload training consisted of training sessions a week of 1–2 sets of 10 exercises of upper-body and core (Day 1) and lower-body (Day 2), during the entire competitive season (27 weeks). Whole body fat mass decreased (-6.3 ± 3.6%, ES = -0.99 ± 0.54) substantially while lean mass increased (2.5 ± 0.8%, ES = 0.25 ± 0.09), with some regional differences. There was a substantial increase in half-squat power output (from 3% to 14%, ES from 0.45 to 1.73) and sprint performance (from 1.1% to 1.8%, ES from -0.33 to -0.44), however performance changes were not correlated with changes in body composition. A combined soccer and eccentric-overload training program was able to promote positive changes in body composition and physical factors relevant to both on-field performance and injury prevention in elite soccer players.

## Introduction

Soccer is considered to be an intermittent sport [1] in which physically demanding, high-intensity actions like sprinting, jumping, and change of direction are important factors for competitive success in both adult [2] and young players [3]. The demand for high-intensity actions has increased over time [4] and nowadays these type of actions are crucial in high-level, competitive soccer, so optimization of those actions and associated factors is essential [5]. In this regard, the central goal of strength-training is to improve the players´ specific and relevant soccer activities inherent to the game [6]. Different resistance training modes have been used to improve physical performance in soccer, such as programs based on traditional exercises with gravitational loads [7], plyometric training [8], ballistic exercises, weight lifting and a combination of different methods [9, 10]. In addition to these well-established strength-training methods, inertial eccentric-overload training programs including flywheel devices have won many adherents in team sports [11] and in soccer players [12–14] over the past years. Flywheel inertial devices provide a source of linear resistance from a spinning disc or cone, which can produce a higher overload and activation in the eccentric phase in comparison with traditional free-weights exercises [15]. As an alternative, or addition, to the more traditional training methods the potential benefits of including inertial eccentric-overload training related to the ability to freely move in the three dimensions for a “more specific” training stimulus [16, 17], the improvement of muscle coordination, the adaptations towards a stronger and faster muscle [18, 19], a reduction in muscle-injury incidence and severity [13, 14] and improvements in strength, linear-sprint speed and change of direction ability [20–22].

However, despite the increased popularity of inertial eccentric-overload training methods in soccer and recent research support [12–14, 20, 21], most of the interventions published to date have investigated the impact of this training paradigm over relative short periods (i.e., 6 to 11 weeks). Moreover, to date no study has evaluated the impact of systematic inertial eccentric-overload training on body composition (BC). In this regard, BC is an important fitness component, relevant to the soccer player´s physiological status as well as performance [23]. Therefore, is very common that professional soccer players asses changes in BC several times over the season to determine the effectiveness of training and nutritional interventions, since changes in body composition may adversely affect on soccer performance and injury prevention [23–25]. For instance, an excess of adipose tissue will load the player with useless extra weight [26]. This would likely contribute to greater energy expenditure during a match and impaired performance in terms of power and acceleration [2, 27]. Moreover, fat-mass (FM) is inversely related to aerobic capacity, athlete’s power-to-weight ratio and thermoregulation, and lean muscle mass strongly contributes to strength and power performance [28]. Thus, while there are not clear standards on what would be the “ideal” BC for a soccer player, practitioners are likely to search for relatively low levels of fat mass [23] and the maintenance of the adequate skeletal muscle mass compatible with the locomotor (e.g., sprints, decelerations, changes of direction and high-intensity and overall running volume) demands imposed by soccer. In recent years, dual-energy X-ray absorptiometry (DXA) has been employed to accurately assess BC in terms of bone mineral content (BMC), bone mineral density (BMD), skeletal muscle mass (SM), fat free soft tissue mass (FFSTM) and FM. To date, several studies [29–33] employed DXA to assess BC in soccer players. An interesting feature of DXA is its ability to yield BC data at the whole body as well as regional level [33].

To our knowledge, there is no previous research that has evaluated the effects of an inertial eccentric-overload training program on BC in soccer players. In addition, there is no information on the effect of a prolonged (an entire competitive season) of inertial eccentric-overload training on strength and physical performance in soccer players. Therefore, the aim of the present study was to describe the changes in BC, strength and sprint performance in response to an entire competitive season of football training supplemented with 2 inertial eccentric-overload training sessions a week in young male elite professional soccer players.

## Methods

### Subjects

This study involved a group of 14 healthy male professional soccer players belonging to the reserve squad of a Serie A club in Italy. All the recruited players were between the ages of 16 and 19 years (17.5 ± 0.8 y; 180.0 ± 6.1 cm; 70.6 ± 5.3 kg; 21.8 ± 1.4 BMI), and some of them also played during the season with the first team (n = 3) and their national team (n = 8). All the players trained ~ 9 hours of soccer training plus 1 or 2 competitive matches per week. To be eligible for the study, players were required to meet the following criteria: i) to have a current professional contract with the club, ii) to be injury free at the moment of the initial assessment, iii) to have completed >85% of the strength training sessions, and iv) not being regularly training with the first team. All the players (or tutor for players under 18) were carefully informed about the experiment procedures and about the potential risk and benefits associated with participation in the study, and signed an informed consent document before being included in the study. The local Institutional Research Ethics Committee (Qatar Antidoping Lab, Doha, Qatar) approved this study, in accordance with the current national and international laws and regulations governing the use of human subjects (Declaration of Helsinki II).

### Experimental design

The presented study used a controlled repeated-measures research design to investigate the BC, strength and linear sprint changes in response to an entire season of football training supplemented with 2 inertial eccentric-overload training sessions a week in young male professional soccer players. Across the season, players had 5–6 training sessions a week, with an average duration of 80 min (from 45 min sessions, to 100–120 min sessions where the inertial eccentric-overload training sessions where included before field training). Whole body and regional composition was evaluated by DXA at the beginning of the competitive period (September) and at the end of the season (May). A lineal sprint test, and a lower-limb power test in half-squat with 2 different weights (30 and 40 kg) were also administered at the beginning and at the end of the competitive period. The sprint test was performed on an artificial turf field in the training ground (not in their usual pitch), whereas the power test was carried out in the gym. All players were familiar with the procedures of both tests and were asked not to perform any strenuous exercise ~ 48 h before the 2-days testing sessions. Each performance test was performed on separate days. The performance tests were carried out at the beginning of the training session and after a standardized warm-up. All of tests were performed at the same time of the day and in the same order: first day of the week (and after a day of rest) the sprint test and the second day the lower-limb power test. The DXA was also performed 2–3 hours before the start of the training session in those 2-days testing.

### Anthropometric and body composition analysis

Body mass was taken at the nearest 0.1 kg with an electronic scale (OHAUS Corp., Florham Park, NJ). Stature was measured with a stadiometer (Seca 213, Hamburg, Germany) at the nearest 0.5 cm. Body composition (FFSTM, FM, BMC and BMD) was measured from whole body and regional scan by means of DXA using a total body scanner (Hologic QDR Series, Delphi A model, Bedford, MA, USA) with analysis performed using Hologic APEX 13.3:3 software version, and according to the manufacturer’s procedures. The DXA was calibrated with phantoms as per manufactured guidelines each day before measurements. The participants assumed a stationary, supine position on the scanning table, with hands level with the hips and feet slightly apart. All scanning and analyses were performed by the same operator to ensure consistency and in accordance with standardised testing protocols that are recognised as best practise [34–36]. As in a previous study, whole-body data are reported herein as total body less head (TBLH) [33]. Scans were performed in the morning before training. Measurement sessions were taken in September (at the beginning of the competitive period) and in May (at the end of the competitive season). DXA SM prediction formula [37, 38] was also evaluated in the players.

### 40-m linear sprint test

Subjects were assessed over a 40-m linear sprint test with split times in 10-m (T10-m), 30-m (T30-m) and 40-m (T40-m). The 40-m sprint test was conducted outdoors with suitable weather conditions (i.e., sunny and no wind) on an artificial turf field. Sprint time was recorded with photoelectric cells (Racetime2, Microgate, Bolzano, Italy). The front foot was placed 0.5-m before the first timing gate, and players started when ready eliminating reaction time. Subjects were given 2 practice trials performed at half speed after a thorough warm-up to familiarize them with the timing device. Two trials were completed, and the best performance trial was used for the subsequent statistical analysis. Two minutes of passive rest were permitted between 40 m trials. Players’ acceleration was defined as their first 10-m sprint time (T10-m), and maximal sprint speed as the running speed attained during the 30-40-m split time [39].

### Lower-limb power test

The lower-limb power performance was determined as the highest mean propulsive power that could be lifted through the full range of motion of a half-squat (HS) exercise (unilateral and bilateral), with correct technique in two different weights (30 and 40 kg). Participants performed the HS from a fully extended position starting with shoulders in contact with the bar. On command, the participants performed a controlled eccentric squat to a knee angle of 90° (using a goniometer), followed without pause by a concentric leg extension returning to full extension. Players were required to execute the concentric phase in an explosive manner at the maximal possible velocity. Three repetitions were performed with each load and the best of them according to the criteria of fastest mean velocity was considered for subsequent analysis. The participants first performed the bilateral HS with 30 kg, and then the unilateral HS with both legs (left and right). After this, the same was done with the second load (40 kg). Two minutes of rest was permitted between loads, and 30 seconds between bilateral and unilateral HS. In all the test the trunk was kept as straight as possible and a Certified Strength and Conditioning Specialist conducted this test and checked for correct technique. A Smith Machine was used for all test (Technogym, Cesena, Italy). A dynamic measurement system was also employed (SmartCoach Power Encoder SPE-35, SmartCoach Europe AB, Stockholm, Sweden), that from bar velocity automatically calculated several kinematic parameters (e.g., vertical displacement, vertical velocity and power output, among other variables) of every repetition. This device was has been widely used to evaluate dynamic muscle work and good reliability scores and certified accuracy of 0.52 ± 0.17% on velocity measurements have been reported [40]. Data were recorded with SmartCoach software V5.0.0.20 (SmartCoach Europe AB, Stockholm, Sweden) to calculate the mean propulsive power for each repetition performed during the HS test. As in previous studies, this power output calculated from bar velocity might be overestimated compared with the use of a force platform [41, 42]. Warm-up consisted of a bilateral and unilateral set of 10 repetitions at loads of 10–15 kg.

### Eccentric-overload training

Players were enrolled in a soccer-training programme 5–6 times a week, with an average session lasting ~ 80 minutes and one day of rest (usually Sunday). In addition, the team participated in an official game once a week (typically on Saturday), and had 5 weeks with 2 games (Wednesday & Saturday). Soccer training in the team focused on the development of technical and tactical skills first and then on the improvement of physical capacities. A half-field game or conditioned game lasting 20–30 min was played at the end of the training session twice a week. All the team supplemented the soccer training with a progressive inertial eccentric-overload training program during the entire competitive season (27 weeks). The strength training was divided into three phases: familiarization phase (3 weeks), progression phase 1 (1 set per exercise, 5 weeks) and progression phase 2 (2 sets per exercise, 19 weeks). The training program took place 2 d/wk. During the progression phase 2, the volume did not change but there was a progression in the intensity (load—free weights—and inertias). To determine the individual load (i.e., inertia) to be employed during the intervention, an assessment with 2 different inertia in K Box (0.10 kg/m^2^ and 0.05 kg/m^2^) and 2 different in Versa Pulley (0.19 kg/m^2^ and 0.26 kg/m^2^) was performed. The inertia with which the player achieved higher average speed was using during the training (SmartCoach Power Encoder SPE-35, SmartCoach Europe AB, Stockholm, Sweden). The inertia was individually readjusted during the progression phase 2 in weeks 7 and 14 of 19. During the weeks with 2 games, only one set per exercise was executed. Typically, upper-body and core training (10 exercises) was performed on Monday (after the day off), and lower-body training (10 exercises) was done on Tuesday. Each strength-training session lasted ~30 min. Upper-body exercises were the following: different push-up exercises using suspension training (Kine Dynamics, Tenerife, Spain (Kine)), several pull-up exercises using Kine, push-up + side-plank-leg, side-bridge + trunk rotation exercises, different plank exercises, side bridge + kicking exercises, prone bridge exercises using fit ball or suspension training, different instable exercises seated on fit ball, several functional unilateral push exercises using Versa-pulley (non-gravity dependent inertial pulley device, VP), and several functional unilateral pull exercises using VP. Lower-body exercises were the following: one-leg Yo-Yo Leg-curl (non-gravity dependent flywheel inertial device), several one-leg hip-extension exercises in VP, several lunges using VP, different HS variations in KBox (non-gravity dependent flywheel inertial device), lateral HS in Kbox, Russian belt deadlift, Russian belt deadlift + hip-rotation exercises (5 kg), Russian belt squat + hip-rotation (5 kg), Russian belt squat + hip-extension, and ankle-extension with free-weights or inertial devices using a step. The resting period between each series was ~1 min. The training was performed in the gym, and all the players were carefully instructed before the training, and received a practical demonstration and performed familiarization trials with all the exercises. The treatment was performed before the start of the regular soccer-training session on the field. All training sessions were fully supervised and training diaries were maintained for the group. No additional training was permitted. An example of the training programme followed for the team is showed in Tables 1 and 2.

**Table 1:** An example of typical training schedule for a training week with one game (progression phase 2).

	Monday	Tuesday	Wednesday	Thursday	Friday	Saturday	Sunday
**Team**	Warm-up (15min) Articular mobility and active stretching Proprioceptive and balance training **Strength training** *Upper-body + Core*, *10 exercises (~ 30 min)*: Push-up KINE 2x12, pull-up KINE 2x12 rep, push-up + side-plank-leg 2x10 rep (5 R+5L), side bridge + trunk rotation 2x10 rep (5R+5L), plank-leg-lift 2x20 s (10 s+10 s), side bridge + kicking 2x10 rep, prone bridge in fit ball 2x20 s, seated in fit ball 2x30 s, VP unilateral push 2x16 rep (8R+8L), VP unilateral pull 2x16 (8R+8L) **Field training** Ball possession games, in *numerical inferiority for players who didn’t play* (10 vs 5, 8x2 min), technical work (10 min), conditioned game 10 vs 8 in half pitch (*in numerical inferiority for players who didn’t play)* (20 min), high-intensity running interval training *only for players who didn`t play (6+6 min)*	Warm-up (10min) Articular mobility and active stretching Proprioceptive and balance training **Strength training** *Lower-body*, *10 exercises (~ 30 min)*: 1 leg leg-curl 2x12 rep (6R+6L), 1-leg hip-extension VP 2x16 rep (8R+8L), lunge in VP 2x16 rep (8R+8L), HS in Kbox 2x8 rep, 1-leg lateral-HS in Kbox 2x12 rep (6R+6L), deadlift in RB 2x8 rep, deadlift in RB + rotation 2x 8 (5kg), HS in RB + rotation 2x10 (5kg), HS in RB + hip-extension 2x10, ankle-extension in VP 2x12 rep **Field training** COD training (10 min), ball possession games (2x4 min), small-side-games with GK (4x4 min), game 10 vs 10 in half pitch (20 min), defensive tactical actions (12 min)	Warm-up (20 min) Articular mobility and active stretching Pass exercises **Field Training** Ball possession game in numerical inferiority (2x4 min) Ball possession game with 2 jockers (10 min) Ball possession game in half pitch (3x10 min) Tactical exercises (12 min)	Warm-up (20 min) Articular mobility and active stretching Rondos Games with short sprints **Field training** 11 vs 0 exercises (8 min) Defensive and attack tactical exercises (15 min) Ball possession game in half pitch (15 min) Conditioned game 10 vs 10 in half pitch (30 min)	Warm-up (15 min) - Active stretching and articular mobility - Reaction games **Field training** Tactical training and set pieces (50 min)	Match	Off

**Table 2:** An example of typical training schedule for a week with 2 games during the study (progression phase 2).

Saturday	Sunday	Monday	Tuesday	Wednesday	Thursday	Friday	Saturday
Match	Warm-up (15 min) Articular mobility and active stretching Aerobic training in bicycle or elliptic, foam roller **Strength training** *Lower-body*, *10 exercises (~ 20 min)*: 1 leg leg-curl 1x12 rep (6R+6L), 1-leg hip-extension VP 1x16 rep (8R+8L), lunge in VP 1x16 rep (8R+8L), HS in Kbox 1x8 rep, 1-leg lateral-HS in Kbox 1x12 rep (6R+6L), deadlift in RB 1x8 rep, deadlift in RB + rotation 1x 8 (5kg), HS in RB + rotation 1x10 (5kg), HS in RB + hip-extension 1x10, ankle-extension in VP 1x12 rep Foam roller and posture Contrast water therapy *(only the players who played)* **Field training** Small-side-games with GK (4x4 min), high-intensity running interval training only for *players who didn`t play (6+6 min)*	Warm-up (20 min) Articular mobility and active stretching Rondos **Field training** 11 vs 0 exercises (8 min) Defensive and attack tactical exercises (10 min) Recovery session for *players who played* (Foam roller, posture, massage & contrast water therapy) Small-side-games with GK (3x4 min), high-intensity running interval training only for *players who didn`t play (6 min)*	Warm-up (15 min) - Active stretching and articular mobility - Reaction games **Field training** Tactical training and set pieces (50 min)	Match	Warm-up (15min) Articular mobility and active stretching Aerobic training in bicycle or elliptic, foam roller **Strength training** *Upper-body + Core*, *10 exercises (~ 20 min)*: Push-up KINE 1x12, pull-up KINE 1x12 rep, push-up + side-plank-leg 1x10 rep (5 R+5L), side bridge + trunk rotation 1x10 rep (5R+5L), plank-leg-lift 1x20 s (10 s+10 s), side bridge + kicking 1x10 rep, prone bridge in fit ball 1x20 s, seated in fit ball 1x30 s, VP unilateral push 1x16 rep (8R+8L), VP unilateral pull 1x16 (8R+8L) Foam roller and posture Contrast water therapy *(only the players who played)* **Field training** Small-side-games with GK (3x4 min), high-intensity running interval training only for *players who didn`t play (6 min)*	Warm-up (10 min) Active stretching and articular mobility **Field training** Tactical training and set pieces (30 min)	Match

### Statistical analysis

Data in fis are presented as means ± standard deviation (SD). All data were first log-transformed to reduce bias arising from non-uniformity error. Possible differences or changes within-player in DXA-measured variables and performance test values were analysed for practical significance using magnitude-based inferences by pre-specifying 0.2 between-subject SDs as the smallest worthwhile effect [43]. The standardized difference or effect size (ES, 90% confidence limit [90%CL]) in the selected variables was calculated. Threshold values for assessing magnitudes of the ES (changes as a fraction or multiple of baseline standard deviation) were >0.20, 0.20, 0.60, 1.2 and 2.0 for trivial, small, moderate, large and very large respectively [43]. Quantitative chances of higher or lower changes were evaluated qualitatively as follows: <1%, almost certainly not; 1–5%, very unlikely; 5–25%, unlikely; 25–75%, possible; 75–95%, likely; 95–99%, very likely; >99%, almost certain [43]. A substantial effect was set at >75% [44, 45]. Pearson’s product-moment correlation analysis was also used to investigate the association between all variables. The following criteria were adopted to interpret the magnitude of the correlation (r) between the different measures: ≤ 0.1, trivial; 0.1–0.3, small; > 0.3–0.5, moderate; > 0.5–0.7, large; > 0.7–0.9, very large; and > 0.9–1.0, almost perfect. If the 90% confidence limits overlapped positive and negative values, the magnitude were deemed unclear; otherwise that magnitude was deemed to be the observed magnitude.

## Results

### Changes in whole body composition

**Fig 1:**
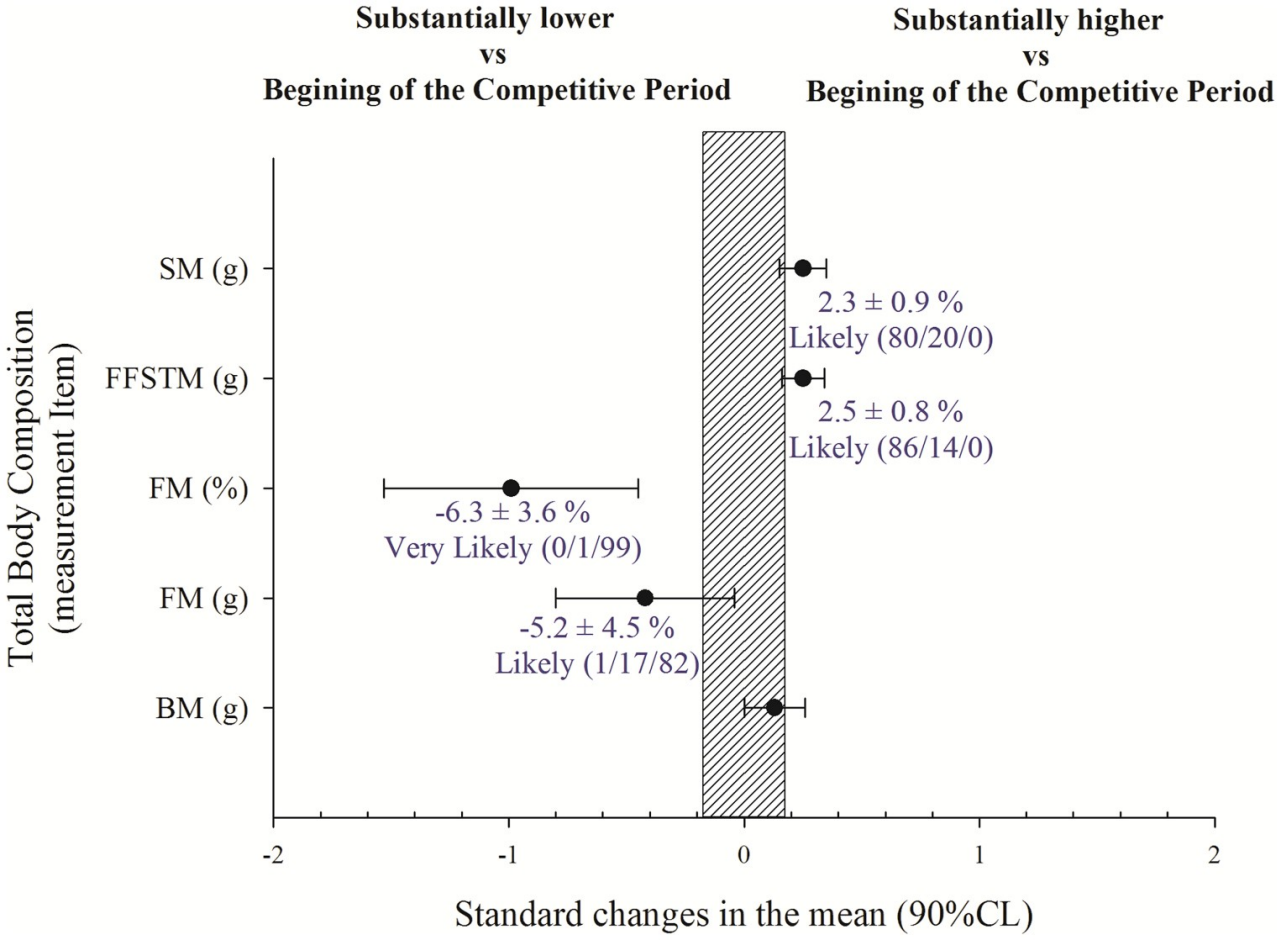
Changes after an in-season training period in skeletal muscle mass (SM), fat free soft tissue mass (FFSTM), fat-mass (FM), and body-mass (BM). Trivial areas were the smallest worthwhile change (see Methods).

Changes in BC after the competitive period are shown in Fig 1. FM and percentage of fat were substantially reduced at the end of the season (ES = -0.42 ± 0.38 and ES = -0.99 ± 0.54, respectively) in comparison with the beginning of the competitive period. SM and FFSTM were substantially higher following the competitive period (ES = 0.25 ± 0.10 and ES = 0.25 ± 0.09, respectively). Statistical analysis showed no changes in BM.

### Changes in arms

Changes in left and right arms after the competitive period are shown in Table 3. Arms-Mass, arms-FFSTM and arms-BMC were substantially increased at the end of the season. BMD increased only in right arm after the competitive period, while a substantial reduction in arm-FM (%) was only observed in left arm.

**Table 3:** Changes in left and right arms after the competitive period. Data are mean ± SD.

*Variables*	Beginning of the Competitive Period	End of the Season	Change in Mean (%)	Standardized differences (90% CL)	Qualitative assessment	
**Left Arm Mass (g)**	4513.5 ± 646.3	4806.1 ± 517.2	6.9 ± 5.0	0.42 ± 0.31	*Likely*	89/11/0
**Left Arm Fat Mass (g)**	695.5 ± 158.6	673.6 ± 140.0	-2.8 ± 10.1	-0.13 ± 0.41	Unclear	9/53/38
**Left Arm Fat Mass (%)**	15.3 ± 2.2	14.0 ± 2.3	-9.1 ± 8.0	-0.58 ± 0.48	*Likely*	1/8/91
**Left Arm FFSTM (g)**	3817.9 ± 530.4	4132.5 ± 436.7	8.6 ± 4.9	0.55 ± 0.31	*Very Likely*	97/3/0
**Left Arm BMC (g)**	200.4 ± 27.9	216.0 ± 17.7	7.9 ± 4.4	0.52 ± 0.28	*Very Likely*	97/3/0
**Left Arm BMD (g∙cm**^**-2**^**)**	0.83 ± 0.06	0.83 ± 0.06	1.2 ± 1.7	0.16 ± 0.23	Unclear	39/60/1
**Right Arm Mass (g)**	4653.3 ± 439.3	4972.6 ± 504.1	6.7 ± 5.7	0.68 ± 0.54	*Likely*	93/6/1
**Right Arm Fat Mass (g)**	790.4 ± 119.7	852.1 ± 120.0	8.0 ± 9.7	0.48 ± 0.57	*Likely*	80/17/3
**Right Arm Fat Mass (%)**	17.0 ± 2.6	17.1 ± 1.7	1.2 ± 7.8	0.04 ± 0.48	Unclear	28/52/20
**Right Arm FFSTM (g)**	3862.9 ± 408.9	4120.6 ± 427.4	6.6 ± 6.0	0.59 ± 0.51	*Likely*	90/9/1
**Right Arm BMC (g)**	203.2 ± 27.3	215.1 ± 28.6	5.9 ± 3.4	0.41 ± 0.23	*Likely*	93/7/0
**Right Arm BMD (g∙cm**^**-2**^**)**	0.83 ± 0.05	0.85 ± 0.06	2.0 ± 1.3	0.32 ± 0.20	*Likely*	85/15/0

**NOTE:** FFSTM = Fat Free Soft Tissue Mass; BMC = Bone Mineral Content; BMD = Bone Mineral Density; CL = Confidence Limits.

### Changes in legs

Changes in left and right legs after the competitive period are shown in Table 4. Legs-FM (%) was substantially reduced at the end of the season, while there were no changes in legs-Mass and legs-FFSTM. Leg-BMC and leg-BMD were substantially increased in the right leg, while a possible increase was observed in the left leg.

**Table 4:** Changes in left and right legs after the competitive period. Data are mean ± SD.

*Variables*	Beginning of the Competitive Period	End of the Season	Change in Mean (%)	Standardized differences (90% CL)	Qualitative assessment	
**Left Leg Mass (g)**	13154.6 ± 1267.1	13126.7 ± 1464.7	-0.4 ± 1.9	-0.02 ± 0.18	Unclear	2/93/5
**Left Leg Fat Mass (g)**	2219.1 ± 395.5	2104.8 ± 460.3	-5.9 ± 8.0	-0.27 ± 0.40	*Possibly*	3/35/62
**Left Leg Fat Mass (%)**	16.8 ± 2.0	15.9 ± 2.2	-5.5 ± 7.3	-0.41 ± 0.49	*Likely*	2/20/77
**Left Leg FFSTM (g)**	10935.9 ± 1010.2	11018.9 ± 1093.7	0.7 ± 1.9	0.08 ± 0.19	Unclear	13/85/1
**Left Leg BMC (g)**	609.5 ± 69.4	627.0 ± 77.9	2.7 ± 2.1	0.23 ± 0.17	*Possibly*	63/37/0
**Left Leg BMD (g∙cm**^**-2**^**)**	1.38 ± 0.08	1.41 ± 0.08	1.5 ± 1.7	0.24 ± 0.27	*Possibly*	60/39/1
**Right Leg Mass (g)**	13784.1 ± 1343.0	13681.9 ± 1477.2	-0.8 ± 2.5	-0.07 ± 0.24	Unclear	3/79/17
**Right Leg Fat Mass (g)**	2533.2 ± 399.4	2392.6 ± 453.6	-6.0 ± 8.6	-0.33 ± 0.49	*Possibly*	4/29/68
**Right Leg Fat Mass (%)**	18.3 ± 1.8	17.4 ± 2.0	-5.2 ± 6.4	-0.48 ± 0.58	*Likely*	3/17/80
**Right Leg FFSTM (g)**	11250.9 ± 1043.0	11289.3 ± 1119.7	0.3 ± 1.7	0.03 ± 0.17	Unclear	5/93/1
**Right Leg BMC (g)**	598.5 ± 74.6	622.5 ± 76.0	4.0 ± 2.3	0.30 ± 0.18	*Likely*	83/17/0
**Right Leg BMD (g∙cm**^**-2**^**)**	1.36 ± 0.08	1.40 ± 0.09	3.0 ± 1.5	0.47 ± 0.24	*Very Likely*	97/3/0

**NOTE:** FFSTM = Fat Free Soft Tissue Mass; BMC = Bone Mineral Content; BMD = Bone Mineral Density; CL = Confidence Limits.

### Changes in trunk, pelvis, spine and ribs

Changes in trunk, pelvis, spine and ribs after the competitive period are shown in Table 5. Trunk-FM (%) was substantially reduced at the end of the season, while a substantial increase was observed in trunk-FFSTM, trunk-BMC and trunk-BMD. BMC and BMD were increased in pelvis and in thoracic spine, while in the lumbar spine and ribs there were no changes.

**Table 5:** Changes in trunk, pelvis, thoracic spine, lumbar spine and lumbar rib after the competitive periods. Data are mean ± SD.

*Variables*	Beginning of the Competitive Period	End of the Season	Change in Mean (%)	Standardized differences (90% CL)	Qualitative assessment	
**Trunk Mass (g)**	31168.9 ± 3110.3	31695.9 ± 3397.5	1.6 ± 2.3	0.16 ± 0.21	Unclear	36/63/1
**Trunk Fat Mass (g)**	4798.5 ± 451.7	4447.2 ± 579.2	-7.7 ± 5.1	-0.72 ± 0.48	*Very Likely*	0/3/96
**Trunk Fat Mass (%)**	15.4 ± 1.0	14.0 ± 1.0	-9.1 ± 3.8	-0.48 ± 0.58	*Almost Certainly*	0/0/100
**Trunk FFSTM (g)**	26370.4 ± 2744.0	27247.8 ± 2920.7	3.3 ± 2.1	0.30 ± 0.19	*Likely*	81/19/0
**Pelvis BMC (g)**	435.7 ± 81.7	463.6 ± 95.9	6.1 ± 2.5	0.32 ± 0.15	*Likely*	91/9/0
**Pelvis BMD (g∙cm**^**-2**^**)**	1.54 ± 0.21	1.60 ± 0.21	3.8 ± 1.4	0.27 ± 0.11	*Likely*	87/13/0
**Thoracic Spine BMC (g)**	97.5 ± 18.0	106.6 ± 20.7	9.1 ± 11.5	0.47 ± 0.57	*Likely*	79/18/3
**Thoracic Spine BMD (g)**	0.83 ± 0.11	0.88 ± 0.14	5.7 ± 3.9	0.42 ± 0.31	*Likely*	88/12/0
**Lumbar Spine BMC (g)**	82.9 ± 25.7	80.9 ± 13.8	0.0 ± 8.8	-0.07 ± 0.32	Unclear	8/68/25
**Lumbar Spine BMD (g∙cm**^**-2**^**)**	1.22 ± 0.19	1.23 ± 0.22	1.0 ± 3.6	0.07 ± 0.24	Unclear	18/78/3
**Left Rib BMC (g)**	91.5 ± 23.2	88.0 ± 10.6	-1.9 ± 12.5	-0.14 ± 0.46	Unclear	11/48/42
**Left Rib BMD (g)**	0.69 ± 0.07	0.67 ± 0.05	-1.6 ± 3.1	-0.16 ± 0.26	Unclear	2/58/40
**Right Rib BMC (g)**	90.2 ± 19.3	86.2 ± 16.7	-3.9 ± 12.7	-0.19 ± 0.53	Unclear	11/40/49
**Right Rib BMD (g∙cm**^**-2**^**)**	0.67 ± 0.07	0.67 ± 0.08	0.1 ± 6.0	0.02 ± 0.53	Unclear	28/49/24

**NOTE:** FFSTM = Fat Free Soft Tissue Mass; BMC = Bone Mineral Content; BMD = Bone Mineral Density; CL = Confidence Limits.

### Changes in performance tests

**Fig 2:**
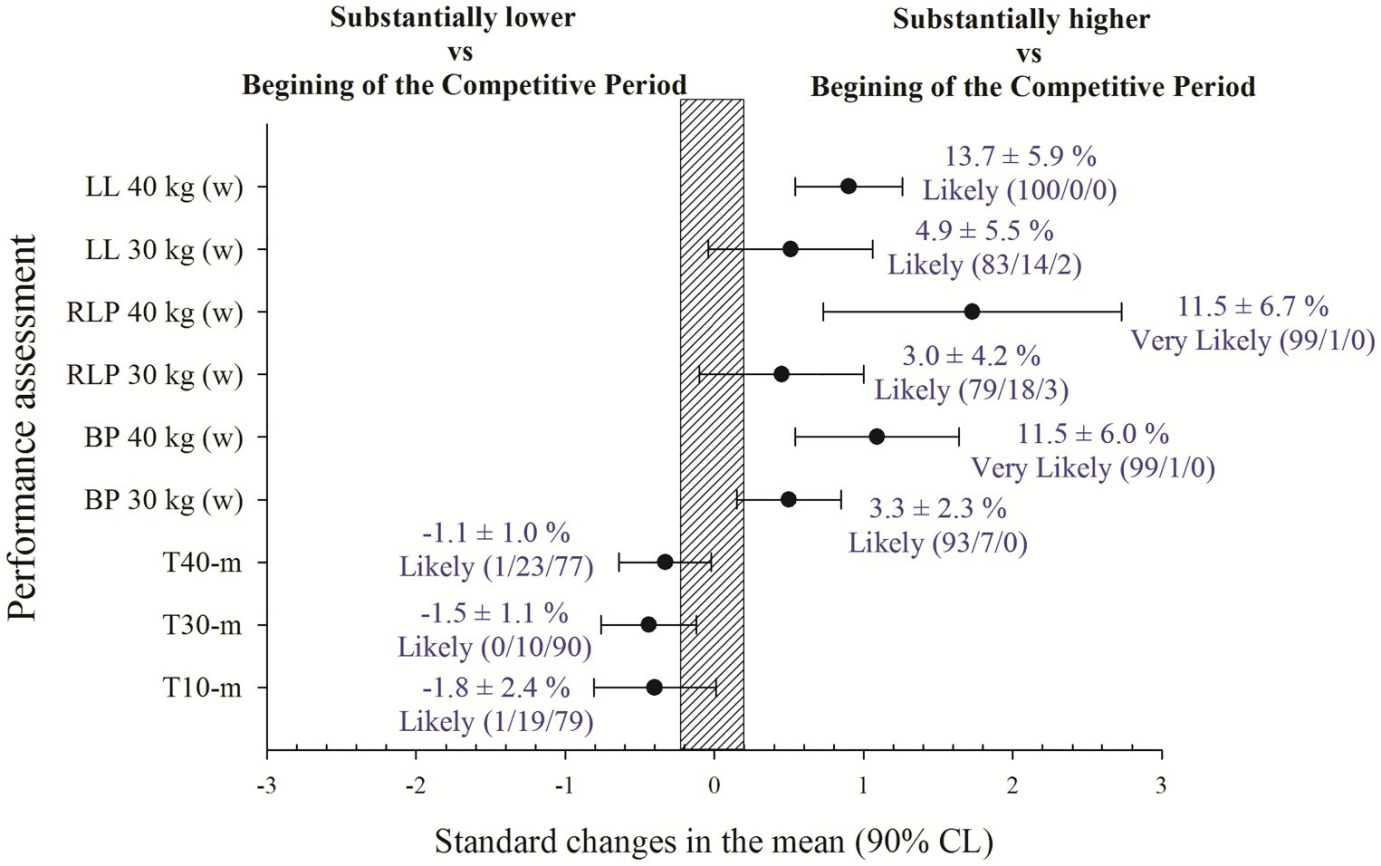
Changes after an in-season training period in mean propulsive power with left leg 40 kg (LL 40 kg), mean propulsive power with left leg 30 kg (LL 30 kg), mean propulsive power with right leg 40 kg (RLP 40 kg), mean propulsive power with right leg 30 kg (RLP 30 kg), mean propulsive power with both legs 40 kg (BP 40 kg), mean propulsive power with both legs 30 kg (BP 30 kg), 40-m sprint time (T40-m), 30-m sprint time (T30-m), and 10-m sprint time (T10-m). Trivial areas were the smallest worthwhile change (see Methods).

Performance tests results are shown in Fig 2. Sprint times were substantially reduced after the in-season period (ES from -0.33 to -0.44). Bilateral and unilateral power was substantially higher with 30 and 40 kg at the end of the season in comparison with the initial test (ES from 0.45 to 1.73).

### Relationships between the different parameters evaluated

When data were pooled, players’ bilateral relative power output with 30 kg was large correlated with sprint times (from r = -0.50 in T10-m to r = -0.58 in T40 m), while with 40 kg was moderate correlated (from r = -0.32 in T10-m to r = -0.45 in T40-m) with sprint times. Unilateral left relative power was large and very large correlated with sprint times (from r = -0.68 in T10-m to r = -0.76 in T40-m with 30 kg, and from r = -0.61 in T10-m to r = -0.67 in T40-m with 40 kg, respectively). Unilateral right relative power was moderate and large correlated with sprint times (from r = -0.55 in T10-m to r = -0.57 in T40-m with 30 kg, and from r = -0.42 in T40-m to r = -0.55 in T10-m with 40 kg, respectively). There were unclear relationships between DXA-measured variables and performance tests.

### Individual relationships between changes in body composition and performance

There were unclear relationships between DXA-measured changes in BC and changes in HS power or lineal sprint. Unilateral left relative power changes were large and very large correlated with improvements in T10-m (r = -0.66 (-0.87; -0.23)), T20-m (r = -0.85 (-0.95; -0.61)), T30-m (r = -0.92 (-0.97; -0.78)) and T40-m (r = -0.88 (-0.96; -0.68)).

## Discussion

The present study analyzed the effects of combined football and eccentric-overload training on BC, power and sprint performance in elite male soccer players. The main findings of the present study were: a) whole body fat mass (total and %) decreased substantially while lean mass increased during the competitive season; b) arms, trunk and legs fat mass decreased, arms and trunk lean muscle mass increased while legs lean mass was maintained during the competitive season; c) sprint performance and HS power output was substantially improved, especially with the higher load (40-kg); d) BC changes were not related with performance (either HS power output or sprinting) changes.

BC in soccer players is likely to change during the course of the competitive season as a result of training, competition and/or diet [46]. In the group of football players investigated in the present study, DXA-derived measurements showed a whole-body reduction in absolute (-5.2%) and relative %FM (-6.3%) during the competitive period. The relative FM (%) at regional level (arms, legs and trunk) substantially decreased at end-season too, and FM reductions was similarly distributed in the trunk, upper and lower body. Reductions in FM, assessed by DXA, were also found by Milanese et al. [33] in elite professional players (Calcio Serie A) between pre-season *vs*. in-season, while within the season an increase in lower-limb and trunk FM were reported. Carling & Orhant [47] found similar results in a study with elite professional soccer players of the French League 1, although in this case using skinfolds measurements. The results of that study [47] showed that whole-body FM (%) decreased after the pre-season training but did not change between the end of pre-season and mid-season, while a significant increase in FM (%) was found at the end of the season with the final value being similar to the preseason value. These authors suggest that this finding can be explained by the tuning down of training intensity towards the end of the season [33]. This was contrary to our findings in the present study. Albeit speculative, the proposed eccentric-overload training program supplementing soccer training could have influenced the results of the present study, in conjunction with the high-intensity of the training sessions until the end of the competitive season [48–50], since the team was fighting until the last games to win the domestic league.

Whole-body data showed substantially increases in SM (2.3%) and FFSTM (2.5%) during the competitive period. Contrary to our results, using also DXA, increases in FFSTM were reported with elite professional soccer players at both mid- and end- season in comparison with pre- season, while no changes were found within the competitive period [33]. Similarly, Carling & Orhant [47] using skinfolds measurements, reported no changes in FFSTM during the in- season period. These differences between those previous studies [33, 47] and current findings might be related to the different global (football + additional contents) training stimulus and/or age of the players. When FFSTM was evaluated by body segments, increases were detected in the arms and trunk regions, while lower limb FFSTM remained unchanged. Similar results were showed by Milanese et al. [33] at both mid- and end- season, but in comparison with pre- season. The substantial increase in upper-body (arms + trunk) FFSTM found in the present work caused by the upper-body and CORE neuromuscular training is an interesting finding, since preventive neuromuscular training programs with strength and proximal control training demonstrated the greatest prophylactic effects on ACL injury risk reduction [51], and recent studies identified a link between proximal segment control and knee joint injury [52, 53]. In addition, players who sustained severe ligamentous knee injuries demonstrated greater deficits in trunk neuromuscular control [52]. Thus, upper-body and core strength and control can be regarded as an important quality in professional football, being an integral part of an effective injury prevention program [54, 55]. In addition, upper-body and core strength and control may affect performance by facilitating abilities such as sprinting, jump, agility or change of direction, having a high impact in all types of duels and contact actions during match-play. The fact that lower limb FFSTM remained unchanged over the competitive period appear to suggest a potential incompatibility between the almost daily heavy metabolic and muscular load imposed by the soccer training and competition and the eccentric-overload strength training proposed. However, the maintenance of lower limb lean-mass and concurrent improvement in lower body power output and sprinting performance observed suggests the suitability of the present combination of soccer and strength training methods, promoting skeletal muscle adaptations in terms of strength and power as in previous studies [56], but without changes in size in our case. Future studies should investigate the compatibility of different strength training paradigms with soccer training.

Changes in BMC and/or BMD during a competitive season have been shown in different sports [57, 58]. Several investigations have shown that soccer practice is associated with increased BMC and/or BMD in comparison to physically active controls [29, 32], but only Milanese et al. [33] investigated changes in bone characteristics across the competitive season in professional soccer players. They showed that regional BMC and BMD generally decrease from pre- to mid-season, and increase above baseline at end-season (except for lower limbs). In contrast to this, our results showed that at all regional levels (arms, legs, pelvis and thoracic-spine) BMC and BMD increased at end-season in comparison with the beginning of the competitive period. These data suggest that exposure to our current strength training and soccer training and competitive activity was beneficial to bone strength and remodelling over a competitive season. Overall, despite the positive impact that the global training stimuli proposed in the present study had on BC, no meaningful associations where found between changes in BC and HS power output and sprinting performance. These results suggest that BC and physical performance should be considered as different “fitness” factors in soccer.

Power output during the exercises tested in the current study (i.e., bilateral and unilateral half-squat) substantially improved during the competitive period. The magnitude of the changes (ES = 0.45–0.50) in power output with the lower load (i.e., 30 kg) was similar to the effect size (ES = 0.42) previously reported in a meta-analysis [59]. However, the changes observed with the highest load (i.e., 40 kg) (ES = 0.90 to 1.73) were substantially greater. These results are within the range of the improvements in 1RM half squat (ES = 1.34) and concentric mean power in half squat (ES = 1.41) recently reported in team-handball players after only 7-w of eccentric-overload training [60]. Thus, it seems that eccentric-overload training has the ability to improve high-loads strength and power output. Albeit a direct 1RM comparison cannot be made with current data, considering the linearity between improvements in concentric mean power and 1RM [61, 62], it can be speculated that eccentric-overload training might have the ability to improve high-loads strength and power output. In addition to HS power output, linear sprint performance was also enhanced (ES = 0.30 to 0.42). Collectively, these results are within the range of those found after different short-term eccentric-overload training programs in different team-sports players (ES = 0.10 to 0.95) [11–14, 21, 60]. Therefore, it seems that eccentric-overload training irrespective of the combination of exercises can be use to optimize linear sprint with soccer players.

This work has some limitations that should be acknowledged. First, we have evaluated professional players from an elite professional club during a competitive full season, and this representative sample has not allowed us to have a control group. Second, the determinant influence of diet on BC was not controlled exhaustively throughout the entire season. It would be interesting for the next investigations to have complete control on these variables.

In conclusion, a 2 d/w strength-training program supplementary to the soccer training sessions in elite professional soccer players may can induce a FM reduction, an increase in FFSTM, BMC and BMD at both whole-body absolute and regional level across the competitive season. In addition, the strength-training program twice a week will also help to improve on elite soccer players power and sprint performance. Although these results must be considered not yet conclusive, they could be important information for coaches, especially during the training prescription and control of physical performance in elite soccer players.
